# Effect of light, gibberellic acid and nitrogen source on germination of eight taxa from dissapearing European temperate forest, *Potentillo albae*-*Quercetum*

**DOI:** 10.1038/s41598-017-13101-z

**Published:** 2017-10-24

**Authors:** Jeremi Kołodziejek, Jacek Patykowski, Mateusz Wala

**Affiliations:** 1 Department of Geobotany and Plant Ecology, Faculty of Biology and Environmental Protection, University of Lodz, Lodz, 90-237 Poland; 2 Department of Plant Physiology and Biochemistry, Faculty of Biology and Environmental Protection, University of Lodz, Lodz, 90-237 Poland

## Abstract

Little is known about how light affects seed germination and revegetation of species of thermophilous oak forest. To reveal this relationship effects of white, red, far-red irradiations and dark incubation on germination of eight *Potentillo albae*-*Quercetum* taxa were examined. Attempts were also made to evaluate the influence of gibberellic acid and different nitrogen sources on the germination characteristics. Interaction between light and nitrogen was also studied. Freshly matured seeds of all taxa germinated very poorly, indicating presence of primary dormancy. Germination rates increased after wet-stratification treatment and were low in darkness. The highest concentration of the nitrogenous solutions that resulted in high germination level was 10 mM, whereas higher concentrations had a negative effect. Nitrate had the strongest influence which can be proved by a ‘gap detection’ mechanism for gaps in the vegetation. Far-red and red irradiation showed antagonistic effect on seed germination. There was a decrease in germination when far-red was followed by red and an improvement when red was followed by far-red treatment. Under red light, gibberellic acid enhanced germination of positively photoblastic taxa. It was concluded that light factor, associated with vegetation gaps, was the most important signal stimulating germination of the studied taxa.

## Introduction

*Potentillo albae*-*Quercetum* Libb. 1933 (*Quercetalia pubescenti-petraeae* order) is a type of thermophilous oak forest occurring farthest north and covering within its range southern Germany, Austria, the Czech Republic, Slovakia, Hungary, south-western Russia and Poland^1,2^. This type of forest occurs on flatland, gentle sunny slopes or in shallow depressions. The habitat is considered a priority type (9110*)^3^.

The *Potentillo albae-Quercetum* communities include forests with tall canopy. The shrub layer is usually developed, although its cover is low^2^. Abundant light reaches the ground as a result of both the spacing of the oaks and the high transmission by their canopy. The light-demanding species that dominate the herb layer are found in dry grasslands (from *Festuco-Brometea*), brushwood (from *Rhamno-Prunetea*) and in wet meadows (from *Molinio-Arrhenatheretea*). The lowest level of this herb layer is composed of species with low light requirements (from *Fagetalia*)^1,4^.

Dynamic tendencies of phytocoenoses observed in thermophilous oak forests indicate urgent need to continue active protection in order to stop the process of transformation of thermophilous oak forests to closed oak-hornbeam forest^1^. According to previous studies^5^, it is well established that understanding of vegetation dynamics requires a combination of intensive field studies (phenology, mortality, seed bank characteristics) and experimental approaches (germination characteristics of the seeds). Knowledge of the factors regulating germination of helio- and thermophilous species of oaks is fragmentary.

Within the oak forest the encroachment of *Coryllus avellana* L.*, Carpinus betulus* L. and *Robinia pseudoacacia* L. undergrowth in forest patch margins has been a widespread phenomenon in Poland. The invasion of them caused considerable deterioration in light conditions of the ground layer, as deduced from a decrease in Ellenberg’s light index values. This results in progressing elimination of heliophilous species^1,4,6^.

The amount of light affecting undergrowth is one the principal conditions controling plant survival and growth. Under open conditions, red (R) light (650–700 nm) predominates over far-red (FR) wavelengths (700–750 nm) with the ratio of R to FR (R/FR) light energy averaging between 0.2 and 1.2 on a clear day^7,8^. As sunlight filters through the canopy, its spectral distribution is changed because of selective spectral absorption of leaves. It is considered that R light radiation is almost fully absorbed by leaves and green light and FR are largely transmitted^9^. Hence, the canopy-filtered light is rich in FR and poor in R. Unfiltered daylight has a typical R/FR ratio of about 1.2 and leaves of canopy may reduce this value to 0.2–0.3, depending on the leaf area index^10^. As a consequence, R/FR ratio increases with gap size and decreases with foliage or litter density^11^. The inhibitory effect of light filtered through leaves on germination is a result of a decreased R/FR ratio which effects the phytochrome system of imbibed seeds^7,10,12^. Most positively photoblastic seeds are inhibited by low R/FR ratio of the canopy-filtered light.

Seed germination is a process controlled by both internal properties of seeds and by an array of environmental factors including light quality, soil moisture availability, alternating temperature and nitrate ions^10,13,14^. It is also known that germination is under control of two phytohormones which act antagonistically: gibberellins and abscisic acid. Production of gibberellins induces germination, while accumulation of abscisic acid negatively regulates this process and is responsible for dormancy^15,16^. Recently it has been found that reduction of the abscisic acid level is controlled in a nitrate-dependent manner by proteins which are transcription factors binding to a promoter of a gene coding an abscisic acid catabolic enzyme^17^. Therefore, the optimal form and dose of nitrogen seem to promote germination by lowering the abscisic acid/gibberellins ratio. This proportion can be also altered by induction and/or inhibition of gibberellins biosynthesis. Metabolism of gibberellins is sensitive to changes of environmental factors, including light quantity and quality (e.g. changing R/FR ratio)^18^. Then, alternations are detected by a phytochrome^19^, converted to an internal signal and transducted to a nucleus, which triggers biosynthesis of gibberellins. Therefore, germination that can be observed in field is the result of the cross-talk between endogenous factors and environmental-triggered stimuli and shows high complexity.

Although various aspect of the autecology of species from *Potentillo albae-Quercetum* have been studied in considerable detail^1,4^, until recently not much attention was given to seed germination. For example, it is not known whether the seeds germinate readily in the dark or if their emergence from the seed bank is restricted to disturbances that expose them to adequate light. Basic information on germination of the presented taxa should add to our understanding its mechanisms in *Potentillo albae*-*Quercetum* species and assist restoration as well as conservation efforts in thermophilous oak forest.

The aim of this study was to test the hypothesis that seeds of the dominating species occuring in herb layer (Table 1) require light to germinate. The following questions were addressed; (1) how do plants respond to different quality of light; (2) can GA_3_ and nitrogen source (KNO_3_, NH_4_Cl or NH_4_NO_3_) promote seed germination; (3) is seed germination photoreversible by FR light after exposure to white or R light.

**Table 1:** List of the studied taxa, their life-form, centre of abundance and environmental conditions preference.

Taxa	Abbreviation	Family	Growth form	Centre of abundance of adult individuals^a^	Ellenberg’s value	
					Light^b^	Nitrogen^c^
*Aquilegia vulgaris* L.	Av	Ranunculaceae	Herb	forests, brushwoods	6	4
*Calamintha acinos* (L.) Clairv.	Ca	Lamiaceae	Herb	brushwoods	7	3
*Digitalis grandiflora* Miller	Dg	Scrophulariaceae	Grass	forests, brushwoods	7	5
*Festuca amethystina* L. subsp*. ritschii* (Hack.) Lemke ex Markgr.-Dann.	Fa	Gramineae	Herb	forests	n.d.	n.d.
*Lychnis viscaria* L. subsp. *viscaria*	Lv	Caryophyllaceae	Herb	xerothermic grasslands, brushwoods	7	4
*Melica nutans* L.	Mn	Gramineae	Grass	forests, brushwoods	4	3
*Serratula tinctoria* L.	St	Compositae	Herb	brushwoods, meadows	6	3
*Stachys officinalis* (L.) Trevisan	So	Labiatae	Herb	brushwoods, meadows	7	3

Plant nomenclature follows literature^50^; life form and Ellenberg’s values describing the effects of ecological conditions on vascular plants^51^. All taxa are common in Poland, except for the *Digitalis grandiflora* and very rare *Festuca amethystina* L. subsp*. ritschii* (Hack.) Lemke ex Markgr.-Dann. ^a^Centre of abundance of taxa^52^. ^b^Light index: the scale ranges from 0 to 9, where the value 9 denotes the highest prevalence of plant. ^c^Nitrogen index: the scale ranges from 3 to 5, where the value 3 indicates nitrogen-poor soils and 5 indicates moderately N-rich soils. n.d. no data.

## Results

### Effect of Different Quality of Light

Seed germination was greatly affected by light quality (Table 2). Exposure to white light significantly (Tukey’s test, *P* < 0.05) increased germination compared to the dark control for all species (Table 3). The white light effect was greatest for the seeds of *Serratula tinctoria* and *Stachys officinalis* which germinated poorly (<16%) in the dark but high germination rate (>96%) was observed in the light. The seeds of other six taxa germinated between 23–43% in dark. R light fully replaced the effects of white light in seed germination for four (*Aquilera vulgaris*, *Calamintha acinos*, *Digitalis grandiflora*, *Festuca amethystina*) taxa. For *Calamintha acinos*, *Digitalis grandiflora* and *Lychnis viscaria* no difference in germination between light and darkness conditions were observed. Seeds of *Calamintha acinos*, *Digitalis grandiflora* and *Festuca amethystina* were highly responsive to R light. Seed germination depended upon the kind of treatment applied at the end. The effect of R light was reversed by FR light and *vice versa*. Seeds of all taxa imbibed in FR light and then exposed to R light had increased germination compared to those not exposed to R light, but this increase was much smaller than that for seeds imbibed in darkness followed by R light. This indicates insensitivity of the seeds to FR light following an initial exposure to R light.

**Table 2:** Germination percentages of seeds exposed to different light treatments.

Irradiation	Germination (%)							
	Av	Ca	Dg	Fa	Lv	Mn	St	So
Dark	23 ± 1e	37 ± 3d	24 ± 1e	22 ± 3d	37 ± 2d	43 ± 3b	14 ± 3d	19 ± 2d
White	92 ± 1a	86 ± 3a	91 ± 3a	91 ± 3a	85 ± 3a	79 ± 3a	97 ± 3a	93 ± 2a
R	66 ± 2b	83 ± 3a	89 ± 3a	84 ± 1b	69 ± 3b	74 ± 3a	68 ± 3b	83 ± 3b
FR	29 ± 2e	42 ± 3d	63 ± 3d	20 ± 3d	55 ± 3c	26 ± 1c	17 ± 2d	18 ± 2d
R + FR	39 ± 1d	57 ± 3c	71 ± 3c	30 ± 3c	27 ± 3e	50 ± 3b	21 ± 3d	21 ± 3d
FR + R	53 ± 2c	77 ± 3b	84 ± 3b	74 ± 3b	50 ± 3c	72 ± 3a	58 ± 1c	79 ± 1c

24-h dark imbibied seeds were exposed for 20 days to 10 min of either light conditions. Germination was observed in dark chamber at 23 °C. Values are mean ± SD (n = 4). Different lower-case letters indicate significant diffences by Tukey’s test with Bonferroni correction in the germination percentages among different light conditions.

**Table 3:** The effect of light (12-h daily photoperiod) or dark in relation to the presence of nitrate and/or ammonium ions (10 mM) on the final germination.

Light treatment	Nitrogen source	Germination (%)							
		Av	Ca	Dg	Fa	Lv	Mn	St	So
Dark	KNO_3_	49 ± 4b	64 ± 5a	53 ± 6b	64 ± 6a	63 ± 4a	68 ± 3a	47 ± 2b	48 ± 1b
Light		97 ± 5a	92 ± 5a	93 ± 4a	95 ± 6a	84 ± 4b	88 ± 6ab	96 ± 2a	97 ± 4a
Dark	NH_4_Cl	43 ± 4b	60 ± 3a	53 ± 1ab	44 ± 1b	60 ± 2a	59 ± 3a	40 ± 2b	41 ± 2b
Light		96 ± 4a	86 ± 3ab	90 ± 6a	93 ± 7a	88 ± 2ab	86 ± 3ab	89 ± 8ab	93 ± 5a
Dark	NH_4_NO_3_	52 ± 4b	65 ± 5a	54 ± 6b	63 ± 5a	62 ± 3a	68 ± 3a	49 ± 2b	50 ± 3b
Light		99 ± 3a	94 ± 5a	98 ± 6a	97 ± 5a	87 ± 6b	92 ± 3a	99 ± 8a	100 ± 6a

Chilled seeds were germinated for 20 days (23 °C). Values are mean ± SD (n = 4). Different lower-case letters indicate significant diffences by Tukey’s test with Bonferroni correction in the germination percentages beetwen taxa.

The PGI values varied between 0.44 and 0.88 (Table 4). Six of the eight studied taxa (rated 6 or 7 for light requirements) occured as adults in large gaps, had a seed mass <1.5 mg, and required light for germination (PGI > 0.56). The most shade-tolerant species tested, *Melica nutans* (Ellenberg’s value of 4), had a seed mass 1.59 mg and germinated better in light than in dark (PGI = 0.44).

**Table 4:** Photo-requirement germination index (PGI) and average seed mass for taxa included in the study.

Taxa	PGI	Seed mass* (mg)
Av	0.65	1.18
Ca	0.56	0.18
Dg	0.73	0.14
Fa	0.75	0.65
Lv	0.57	0.05
Mn	0.44	1.59
St	0.88	1.36
So	0.83	0.44

*The mean seed mass of each taxa was determined by weighing 100 air-dried seeds.

### Effect of Different Type and Concentrations of Nitrogen Source and Chilling

Germination was generally very low in the seeds incubated immediately after harvesting. Maximum promotion of germination of all taxa was obtained with the concentration of 10 mM NH_4_NO_3_ after 16 wk chilling. *Calamintha acinos*, *Festuca amethystina*, *Lychnis viscaria* and *Melica nutans* presented the highest germination. A considerable enhancement of germination was also obtained at 1 mM. Germination of all taxa was again shown to be promoted by KNO_3_ (NH_4_NO_3_ being slightly more effective than KNO_3_). NH_4_Cl had less effect than both NH_4_NO_3_ and KNO_3_ (Table 5).

**Table 5:** Effect of different concentrations of KNO_3_, NH_4_Cl and NH_4_NO_3_ combined with chilling on total germination of the examined taxa.

Chilling period (weeks)	Nitrogen source (mM)	Germination (%)							
		Av	Ca	Dg	Fa	Lv	Mn	St	So
	KNO_3_								
0	0	0 ± 0Ac	0 ± 0Ac	4 ± 0Ac	6 ± 1Ac	0 ± 0Ac	8 ± 1Ac	0 ± 0Ac	5 ± 1Ac
	1	9 ± 1Bb	16 ± 0Ab	9 ± 1Bc	17 ± 1Ab	12 ± 0Ab	14 ± 2Ab	16 ± 0Ab	18 ± 1Ab
	10	19 ± 3Ba	34 ± 3Aa	29 ± 3Aa	34 ± 3Aa	28 ± 3Aa	25 ± 0Aa	31 ± 3Aa	34 ± 3Aa
	25	4 ± 3Ac	6 ± 2A	5 ± 3A	6 ± 2A	8 ± 2Abc	9 ± 0Ac	7 ± 1Ac	10 ± 1Ac
	50	0 ± 0Ac	0 ± 0Ac	1 ± 0Ac	5 ± 1Ac	2 ± 0Ac	2 ± 0Ac	0 ± 0Ac	2 ± 1Ac
16	0	24 ± 2Db	37 ± 2C	25 ± 6D	23 ± 4D	36 ± 2A	41 ± 3B	12 ± 2E	11 ± 1E
	1	28 ± 2Cb	42 ± 3B	31 ± 5C	28 ± 3C	39 ± 5B	52 ± 3A	25 ± 2C	27 ± 2C
	10	49 ± 3Ba	62 ± 3A	42 ± 5B	63 ± 4A	62 ± 3A	69 ± 5A	46 ± 2B	47 ± 2B
	25	19 ± 4ABc	13 ± 2B	17 ± 2AB	14 ± 6B	22 ± 4A	18 ± 3A	16 ± 2AB	12 ± 1B
	50	4 ± 0Ad	0 ± 0A	4 ± 1A	4 ± 0A	4 ± 1A	3 ± 0A	0 ± 0	3 ± 1A
	NH_4_Cl								
0	0	0 ± 0Ad	0 ± 0Ac	4 ± 0Ac	6 ± 1Ac	0 ± 0Ad	8 ± 1Ac	0 ± 0Ad	5 ± 1Ac
	1	18 ± 1Ab	14 ± 0Ab	13 ± 1Ab	16 ± 1Ab	18 ± 0Ab	17 ± 1Ab	14 ± 0Ab	21 ± 1Bb
	10	29 ± 3ABa	26 ± 3Ba	27 ± 0Ba	31 ± 1Aa	35 ± 0Aa	39 ± 4a	25 ± 0Ba	36 ± 1Aa
	25	10 ± 1Ac	6 ± 2Ac	7 ± 3Ac	5 ± 1Ac	10 ± 2Ac	9 ± 1Ac	8 ± 2Ac	9 ± 2 Ac
	50	2 ± 0Ad	2 ± 0Ac	1 ± 0Ac	0 ± 0Ac	0 ± 0Ad	1 ± 0Ad	0 ± 0Ad	0 ± 0Ac
16	0	25 ± 2Bb	38 ± 2Ac	24 ± 2Bc	26 ± 1Bc	37 ± 5Ac	41 ± 3Ab	12 ± 2Cb	11 ± 1Cc
	1	27 ± 2Bb	49 ± 2Ab	29 ± 1Bb	34 ± 1Bb	46 ± 3Ab	48 ± 3Ab	17 ± 2Cb	28 ± 1Cb
	10	43 ± 4Ca	62 ± 7Aa	53 ± 2Ba	45 ± 2Ca	64 ± 5Aa	59 ± 2Aa	40 ± 2Da	43 ± 2Da
	25	13 ± 4Cc	16 ± 3Cd	10 ± 1Dd	8 ± 1Dd	24 ± 2Ad	14 ± 3Bc	6 ± 2Dc	4 ± 2Dd
	50	3 ± 0Ad	1 ± 0Ae	4 ± 1Ae	4 ± 1Ad	2 ± 0Ae	0 ± 0Ad	0 ± 0Ac	0 ± 2Ad
	NH_4_NO_3_								
0	0	0 ± 0Ac	0 ± 0Ad	4 ± 0Ad	6 ± 1Ac	0 ± 0Ad	8 ± 1Ac	0 ± 0Ad	5 ± 1Ac
	1	8 ± 1Cb	24 ± 0Ab	27 ± 1Ab	18 ± 1Bb	17 ± 0Bb	21 ± 2Ab	17 ± 0Bb	19 ± 1Bb
	10	19 ± 3Ba	35 ± 4Aa	34 ± 3Aa	36 ± 3Aa	33 ± 5Aa	38 ± 4Aa	34 ± 5Aa	35 ± 4Aa
	25	11 ± 3Ab	12 ± 3Ac	14 ± 4Ac	16 ± 3Ab	10 ± 1Ac	11 ± 6Ac	9 ± 3Ac	7 ± 3Ac
	50	0 ± 0Ac	1 ± 0Ad	0 ± 0Ad	3 ± 0Ac	1 ± 0Ad	3 ± 1Ad	1 ± 0Ad	2 ± 0Ac
16	0	23 ± 2Bb	37 ± 2Ac	24 ± 6Bb	23 ± 8Bc	36 ± 2Ac	44 ± 3Ac	12 ± 2Cc	12 ± 1Cc
	1	27 ± 2Cb	46 ± 3Ab	29 ± 5Cb	36 ± 3Bb	49 ± 3Ab	55 ± 3Ab	28 ± 2Cb	27 ± 2Cb
	10	52 ± 3Ba	64 ± 3Aa	53 ± 5Ba	62 ± 4Aa	61 ± 3Aa	68 ± 5Aa	49 ± 4Ba	54 ± 2Ba
	25	9 ± 4Ac	14 ± 2Ad	9 ± 1Ac	11 ± 1Ad	12 ± 3Ad	18 ± 3Ad	9 ± 2Ad	14 ± 3Ac
	50	0 ± 0Ad	2 ± 0Ae	0 ± 0Ad	3 ± 0Ae	1 ± 1Ae	3 ± 1Ae	1 ± 1Ae	2 ± 0Ad

Seeds were germinated in dark at 23 °C. Values are mean ± SD (n = 4). Different upper-case letters indicate significant differences by Tukey’s test with Bonferroni correction in the germination percentages between taxa and different lower-case letters in each column with the same chilling period indicate significant differences in the germination percentages of the seeds among different concentration of KNO_3_, NH_4_Cl and NH_4_NO_3_.

In the absence of exogenous N or chilling, only 0–8% of seed germinated. Chilling and addition of either nitrate (NO_3_^−^) and ammonium (NH_4_^+^) significantly enhanced germination. There was also significant interaction between the influence of NO_3_^−^ or NH4^+^ and chilling on germination. In the absence of chilling and after 4 weeks of chilling, germination increased with growing NO_3_^-^ and NH_4_^+^ concentration. Overall, chilled seeds germinated better than non-chilled ones. Chilling stimulated germination in seven of the eight taxa. N level, chilling period and their interaction significantly affected germination (Table 6).

**Table 6:** Results of a three-way ANOVA applied to the germination percentages of seeds incubated at different nitrogen level (0, 1, 10, 25, 50 mM), forms (KNO_3_, NH_4_Cl and NH_4_NO_3_) and chilling periods (0 vs 16 weeks).

Factor	*F* values and significance							
	Av	Ca	Dg	Fa	Lv	Mn	St	So
N level (A)	22.04***	24.34***	23.34***	24.57***	27.67***	24.61***	29.68***	28.55***
N form (B)	21.90*	26.12*	25.06*	27.91*	25.42*	27.86*	24.55*	23.14*
Chilling period (C)	18.45*	18.41*	19.15*	18.99*	14.68*	15.38*	16.27*	18.01*
A × B	14.34**	11.23**	13.21**	12.13**	15.93**	15.27**	11.37**	13.50**
B × C	7.92**	7.02**	8.78**	9.42**	5.74**	7.77**	6.61**	7.98**
A × C	6.46**	7.12**	7.98**	5.46**	4.67**	6.50**	5.47**	6.03**
A × B × C	4.12**	5.65**	4.70**	6.09**	5.56**	4.48**	7.02**	4.59**

*P < 0.05, **P < 0.01, ***P < 0.001 (Tukey’s test with Bonferroni correction).

### Effect of Nitrogen Source Type and Light

Nitrogen added as nitrate ions (KNO_3_), was statistically more effective than that from ammonium ions (added as NH_4_Cl). In addition, germination in the combined presence of the two ions, added as NH_4_NO_3_ solution, was statistically higher than with either one of them (Table 3).

Although both light and exogenous nitrogen alone resulted in statistically significant promotion of germination, the combined presence of these factors was the most inductive. Two-way interactions of N form and light were significant (Table 7).

**Table 7:** Results of a two-way ANOVA applied to the germination percentages of seeds incubated at different nitrogen forms (KNO_3_, NH_4_Cl and NH_4_NO_3_) (each 10 mM) under 12-h daily photoperiod or in continuous dark.

Factor	*F* values and significance							
	Av	Ca	Dg	Fa	Lv	Mn	St	So
N form (A)	21.90*	23.45*	24.85*	22.16*	24.71*	23.11*	22.34*	22.96*
Light (B)	8.36***	9.31***	7.84***	6.45***	9.56***	8.89***	7.98***	8.34***
A × B	18.45**	15.78**	16.71**	15.82**	15.90**	17.33**	16.35**	15.98**

*P < 0.05, **P < 0.01, ***P < 0.001 (Tukey’s test with Bonferroni correction).

### Effect of GA_3_

GA_3_ promoted dark germination of all species. Seeds of all examined taxa given R light irradiation and then imbibed in 100 ppm GA_3_ germinated just as well as dark-treated seeds kept in water. Exposure of imbibed seeds to 48 h FR before the application of GA_3_ prevented germination (Table 8).

**Table 8:** Effect of GA_3_ on germination of red or far red-treated seeds.

Irradiation	GA_3_ (100 ppm)	Germination (%)							
		Av	Ca	Dg	Fa	Lv	Mn	St	So
Dark	+	46 ± 2c	59 ± 3c	48 ± 2c	47 ± 4c	57 ± 5c	66 ± 4bc	38 ± 1c	45 ± 2c
Far red	−	28 ± 2d	41 ± 2d	62 ± 3b	20 ± 2d	53 ± 4c	24 ± 2d	15 ± 1d	15 ± 2d
Far red	+	27 ± 3d	44 ± 3d	61 ± 7b	19 ± 5d	56 ± 3c	23 ± 4d	17 ± 5d	12 ± 3d
Red	−	64 ± 8b	82 ± 5b	90 ± 6a	81 ± 8b	71 ± 6b	75 ± 9b	62 ± 3b	81 ± 4b
Red	+	83 ± 50a	94 ± 8a	99 ± 8a	92 ± 4ab	87 ± 6a	89 ± 5a	79 ± 4a	90 ± 7ab

Values are mean ± SD (n = 4). Different lower-case letters within a column indicate significant differences by ANOVA with Bonferroni correction followed by Tukey’s test in the germination percentages between the taxa. + with phytohormone, − without phytohormone.

## Discussion

Seeds of all studies species sown soon after harvest showed very low germination which indicates presence of primary dormancy. Furthermore, germination rates of all taxa increased after wet-stratification treatment. In nature such a mechanism effectively delays germination until spring. It is consistent with the previous study on the forest species in a temperate region^20^.

It was shown in this study that germination can be induced by short R irradiation applied after 24 h of RF light inhibition. Promotion of seed germination caused by R light was reported for a great number of plant species and is a phenomenon well established in literature^21–23^. For example, germination of *Ruellia tuberose* L.^24^ and *Asteracantha longifolia* (L.) Nees^25^ seeds can be promoted by R light treatment. This promotion can be reversed by a subsequent exposure to FR. Described inhibitory effect of FR expired after 17–20 h of R light treatment. All physiological actions that can be altered by changing R/FR ratio (including germination) are considered as processes controled by phytochrome. Our study showed that Lv and Dg were weakly inhibited by FR while level of inhibition for Av, Ca, Fa, Mn, St and So was moderate. Similar results were obtained for two of these species (Lv and Av) after field studies^23^. It may indicate that phytochrome-mediated regulation of Av, Ca, Fa, Mn, St and So seed germination is tuned to maximalize chance for fast population establishment by germination of banked seeds. It is worth mentioning that germination of some forest woody species (e.g. *Abies alba* L., *Betula pubescens* Ehrh., *P. strobus* L. and *P. sylvestris* L.) is also sensitive to light quality: R light stimulates germination while FR light inhibits this process^26,27^. Elimination of specimens with canopy that strongly affects light spectrum may therefore lead to competition beetwen herbaceous and woody species. It also suggests that many herbs occuring in *Potentillo albae-Quercetum* are able to win competition for resouces due to efficient phytohormone regulation of germination.

Phytochromes are well known to mediate light-promoted germination; they are also known to increase the amount of bioactive gibberellins in seeds^28^. Our results showed that exogenously applied gibberellins promoted dark germination of the studied species. Prolonged FR light pretreatment caused permanent loss of sensitivity to GA_3_. However, it was stated that continuous FR light irradiation delayed gibberellin mediated promotion of germination but did not prevent germination permanently^29^. It was also indicated that different kinds of processes are involved in the biochemical control of germination^30^. This was consistent with studies on *Lactuca sativa* L.^31^.

Nitrates which naturally occurr in soil, can substitute for the light requirement in some cases^32,33^. In this study, four taxa: Ca, Mn, St and So are connected with poor-soil environments and their Ellenberg’s nitrogen index is 3 (Table 1). Up to now little was known about the effects of nitrate concentration combined with light quality on germination. Our results show that germination of these species was enhanced by the lowest concentration (in the range 1–10 mM) of any of the nitrogenous solutions. High concentrations of nitrogen compounds (25–50 mM) resulted in lower germination percentage than controls. This could be related to their ability to colonize soils with low nitrogen concentration. Similar results for seeds soaked in nitrogen solutions as those used in our study have been shown for 10 species from shrubby woodlands in central-western Spain^34^. However, the optimal conditions for seedling growth might not correspond to those for seed germination and seedling survival^10,35^. It is interesting to note that the critical nitrate concentration range for germination induction, observed in laboratory experiment, is spectacularly close to that encountered in natural ecosystems^36^.

Seeds of all examined species need light exposure to complete germination. Only Ca, Mn and Lv germinated >30% in the dark. Based on the white light germination characteristics, the data indicate that many of these species may be able to form a large persistent seed bank. This response, previously shown for many small-seeded temperate species^37,38^, can be seen as an evolutionary adaptive mechanism that prevents seed germination under shaded conditions^10^, as well as under excessively deep soil layers^39^.

The distinct light requirements for seed germination of the studied taxa could be a major factor hampering its natural regenerations. Strong requirements of light suggest germination preference for large vegetation gaps. For forest canopy of temperate forests, areas under canopy openings were found to have higher light intensities as well as higher air and soil temperatures than the surrounding closed forest^40,41^. Therefore, to improve the natural regeneration of *Potentillo albae-Quercetum*, disturbance (e.g., thinning) should be applied to allow more light reaching the understory layer.

A general correlation between seed weight and light requirement for germination has been suggested^37,38,42^. It was found that in temperate forest seed dry mass of 1.5 mg was an approximate cutoff between herbaceous species that are light-dependent and light-independent^39^. Our experiments indicated that seed germination of all 8 studied taxa of *Potentillo albae-Quercetum* was promoted by light. A light requirement for germination was stronger in smaller than in larger seeded species of *Potentillo albae-Quercetum*. PGI decreased with increasing seed mass. Such relationship was reported previously for example for Campanulaceae^42^ and for herbaceous species of northern temperate deciduous forests^43^.

In recent years fast advancing changes in some types of heliophilous oak and oak-pine forests, where there are the best habitat conditions for the studied species, have been observed. The gradual invasion of *C. avellana* and *C. betulus* shrubs is connected with a considerable deterioration of light conditions in the ground layer which results from the closure of the canopy^1^. This variation in vegetation density creates new conditions with altered R/RF ratios of irradiance. Thus, germination of any seed falling under plant canopy (e.g. cover shrubs) could be predominantly inhibited by R/RF ratio which would also contribute to the formation of persistent seed banks of these species^7,8,11^. Germination of any seed falling under a plant canopy may be inhibited by exposure to FR and by lack of R light. Removal of *C. avellana* and *C. betulus* results in greater irradiance of R light than under intact canopies. It could be hypothesized that seedling emergence of the studied taxa may be increased after canopy removal as a result of increased germination of seeds exposed to white and R light. Moreover, the germination of most seeds of the studied taxa occurs in early spring before leaf expansion. The subsequent reduction in light transmittance after leaf expansion would be disadvantageous to growth of seedlings of helio- and thermophilous species.

In many habitats the establishment of seedlings depends on the presence of sites that are clear of vegetation, namely vegetation gaps^12^. Environmental conditions in vegetation gaps often differ considerably, depending on type and size of the gaps, as compared to those of the intact forest^44^. For example, it was found that vine maple gaps, compared with closed canopy of conifer forest, had significantly higher pH values and higher concentrations of Ca, Mg and K in the forest floor^45^. A tendency for lower C/N ratios and higher total N concentrations in the surface mineral soil was also observed. Germination in response to elevated NO_3_^−^ concentrtion in our study can be interpreted as gap formation or as gap-detection mechanisms^10,37^. Some authors stated that nitrates could be a useful indicator of small scale disturbances in forests, since rise in the soil nitrate level can even be observed in single tree-fall gaps^46^. Seedling that rapidly establish in gaps have an advantage over plants that germinate later, when there is a greater competition for resources^10^. The results of this study have important implications for *Potentillo albae*-*Quercetum* restoration programmes. The distinct light requirements for seed germination of the species of *Potentillo albae*-*Quercetum* phytocoenoses could be the most important factor hampering its natural regeneration. In the phytocoenoses of *Potentillo albae*-*Quercetum*, emergence of light-demanding woody species from the seed bank is triggered by disturbance when gaps in litter or plant canopy expose seeds to light or higher R/FR ratio. Therefore, to improve the natural regeneration of phytocoenoses in thermophilous oak forests, disturbances (e.g., thinning) should be applied to allow more light reaching the understorey layer. Moreover, cold stratification breaks dormancy and promotes germination of selected species occurng in temperate forest and this suggests that the examined taxa are specialized to germinate in spring before leaf expansion of canopy. It leads to the conclusion that for many of these species revegetation is strictly connected with seed bank establishment and efficient detection of light condition change.

## Materials and Methods

### Study Site and Seed Collection

Seeds of eight taxa from termophilous oak forest, *Potentillo albae*-*Quercetum*, located in the vicinity of Poddębice, central Poland (51°91′72″N, 18°89′42″E) (Table 1), were collected for all the experiments. The taxa were as follows: *Aquilegia vulgaris* L., *Calamintha acinos* (L.) Clairv., *Digitalis grandiflora* Miller, *Festuca amethystina* L. subsp*. ritschli* (Hack.) Lemke ex Markgr.-Dann., *Lychnis viscaria* L. subsp. *viscaria*, *Melica nutans* L., *Serratula tinctoria* L. and *Stachys officinalis* (L.) Trevisan. The selected species belong to different plant families and are important components of the plant communitiy in the study area. The forest stand is primarily (84%) *Quercus robur* L. (Fagaceae), some of which are 150–200 yr old, with 12% *Pinus sylvestris* L. (Pinaceae) and 4% *Carpinus betulus* L. (Betulaceae). Common species of the shrub layer include *C. betulus*, *Frangula alnus* Mill. (Rhamnaceae), *C. avellana* L. (Betulaceae) and *Q. robur* seedlings (J. Kołodziejek, personal observation).

The study site is located in the transition zone between the temperate oceanic (Atlantic) climate in the west and the moderate continental climate in the east. Mean annual temperature (2000–2010) is 8.8 °C; mean annual precipitation is 570.1 mm, with a maximum in June–July; the average length of the growing season is 210–220 days^47^.

Seed material was collected in the study region between May and October 2015 depending on the time of ripening. In order to obtain representative seed samples of the local populations, mature seeds of each taxa were collected from at least 15 individuals of one single large population and mixed before use. In the laboratory, the seeds were removed from fruits, dried at room temperature and stored in paper bags with relative humidity 40–60% in the dark at laboratory temperature (23 °C) for a maximum of two weeks. By selecting eight species we intended to include typical species of termophilous oak forest occuring under oak tree stand with different seed weights and dispersal strategies, representing a broad variety of families and life forms.

### General Germination Procedures

All germination tests were done in sterile plastic Petri dishes (9 cm diameter) lined with two layers of filter paper (Whatman no. 1) moistened with 3 ml of distilled water (pH 6) or the tested solutions. The Petri dishes were sealed with parafilm to minimize the loss of water. Each experiment consisted of four replicate Petri dishes of 25 seeds for each of the eight species and each treatment. Germination was recorded daily for 20 days. Germinated seeds were removed from the Petri dishes. A seed was considered to have germinated when a radicle had emerged. All manipulations in the dark treatments were done under dim green safe light. Germination percentages were calculated on the basis of the number of viable seeds; dead seeds, which were identified based on their softness and brownish embryo colour, were excluded. Number of dead seeds did not differ statistically among groups and treatments (*P* < 0.05). Then some seeds were stratified in a refrigerator at 5 °C for a 16 weeks to fully break dormancy. This temperature is a standard for testing cold stratification and simulated average winter (5 °C) temperature condition. Non-chilled seeds were stored at 23 °C. The temperature of 23 °C appeared most suitable for optimal germination for herbaceous species from the temperate region^14,48,49^. In addition, this thermoperiod represents the mean daily maximum and minimum monthly temperatures at the Lodz Weather Station during June and July, when most seeds germinate in natural habitat. Fluorescent and incandescent sources of light were used. Light was filtered through 3 mm thick plexiglas filters (locally manufactured).

### Effect of Different Quality of Light

This experiment was performed using the seeds after 16 weeks of stratification at 5 °C prior to germination. Four light treatments plus dark control were used. First, the cold stratified seeds were imbibed in darkness (dishes covered with two layers of aluminium foil) for 12 h on filter paper moistened with 5 ml distilled water. Next, the seeds were exposed to different light treatments: (a) white light provided by a single fluorescent lamp (60 W); (b) R light for 10 min per day obtained by filtering the white light of 100 W incandescent bulb through red plexiglas; (c) FR light for 10 min per day obtained by filtering the white light of 100 W incandescent bulb through blue and red plexiglas; (d) 10 min of R, followed by 10 min of FR per day; (e) 10 min of FR, followed by 10 min of R per day. Dark controls were conducted in absolute darkness. The bulbs were placed 30 cm above the level of the seeds. Germination was observed in a dark chamber at 23 °C for 20 days.

For each taxa, an index of light requirement for germination (photo-requirement germination index; PGI) was derived: PGI = 1 − (FGD/FGL) where FGD is the percentage of germination in the dark and FGL is the percentage of germination in the light. Therefore if all of the seeds germinated in the dark as well as in the light during one day, PGI index would be 0; a value of 1 indicates germination only occurring in the light^37^.

### Effect of Different Type and Concentrations of Nitrogen Source and Chilling

Chilled (16 weeks) and non-chilled seeds were treated with the following nitrogen (N) concentrations: 0, 1, 10, 25, 50 mM N, applied either as KNO_3_, NH_4_Cl or NH_4_NO_3_. Each seed lot was moistened with the appropriate N solution and placed in the dark for the chilling treatment. For the non-chilling treatment, seeds were treated in a similar manner, but chilling (5 °C) for 24 h was substituted for room temperature (23 °C). Seeds were then returned to Petri dishes and incubated in the dark. Germinated seeds were counted under dim green safe light.

### Effect of Nitrogen Source Type and Light

10 mM sources of N were applied as KNO_3_, NH_4_Cl or NH_4_NO_3_ solutions. All taxa were tested under two light conditions (complete darkness and exposure to daylight). Each seed lot was moistened with the appropriate N solution and placed in the dark for the chilling treatment. Next, seeds were transferred to 23 °C and wrapped in aluminium foil for dark treatment or exposed to light for 12 h. Germinated seeds were counted under dim green safe light or in the daylight, respectively.

### Effect of GA_3_

Seeds were first imbibed in 5 ml of distilled water under R or RF light for 48 h. Then the seeds were imbibed either in distilled water or GA_3_ solution (100 ppm) in the dark for 24 h. Procedures with imbibed seeds were carried out under a dim green lamp at 23 °C. The seeds were incubated at 23 °C in continuous darkness.

### Statistical Analysis

Germination percentages were arcsine transformed in order to stabilize variances. Normality was verified with the Shapiro-Wilk test. The data were analysed using one-, two- and three-way ANOVA. Significance of differences amongst treatment means was assessed using Tukey’s multiple comparisons test with Bonferroni correction. All statistical tests were performed using a statistical software package (Statistica, Statsoft USA).

### Data Availability Statement

All data generated or analysed during this study are included in this published article.
